# Power through ‘Us’: Leaders’ Use of We-Referencing Language Predicts Election Victory

**DOI:** 10.1371/journal.pone.0077952

**Published:** 2013-10-23

**Authors:** Niklas K. Steffens, S. Alexander Haslam

**Affiliations:** School of Psychology, University of Queensland, Brisbane, Queensland, Australia; University of Udine, Italy

## Abstract

Leaders have been observed to use distinct rhetorical strategies, but it is unclear to what extent such strategies are effective. To address this issue we analyzed the official election campaign speeches of successful and unsuccessful Prime Ministerial candidates in all 43 Australian Federal elections since independence from Britain in 1901 and measured candidates' use of personal (‘I’, ‘me’) and collective pronouns (‘we’, ‘us’). Victors used more collective pronouns than their unsuccessful opponents in 80% of all elections. Across all elections, victors made 61% more references to ‘we’ and ‘us’ and used these once every 79 words (vs. every 136 words for losers). Extending social identity theorizing, this research suggests that electoral endorsement is associated with leaders' capacity to engage with, and speak on behalf of, a collective identity that is shared with followers whose support and energies they seek to mobilize.

## Introduction

The oratory of great political leaders has been subjected to meticulous analysis by psychologists, linguistics, political scientists, and historians [1]–[5]. This research observes that these leaders tend to use distinct rhetorical strategies. For example, research suggests that successful leaders act as *entrepreneurs of identity* such that their speeches serve to cultivate a sense of ‘us’ that is shared with potential followers [6]–[9]. However, prior research has not established whether political leaders' use of such strategies is actually related to their ability to secure follower endorsement. Here we examine whether successful candidates in national general elections make greater use of we-referencing language than their losing counterparts.

In line with common media portrayals, classical leadership research generally focuses on the (extraordinary) traits and capabilities of individual leaders as “great men” [10]–[14]. In these terms, leaders are understood to be superior beings who succeed because they are different to, and better than, other more ordinary mortals. However, more recent research has shifted focus away from the leader as a great ‘I’ by stressing the importance of followers and the group as a whole to the leadership process [15]–[17]. This places greater emphasis on the ‘we’ of leadership, and is exemplified by work examining the role that a sense of shared group membership plays in allowing leaders and followers to influence each other [18]–[22].

### Leaders' Management of a Shared ‘we’

In this regard, social identity theory asserts that individuals are able to think and act not just as ‘I’ and ‘me’ (in terms of personal identity) but also as ‘we’ and ‘us’ (in terms of social identity) [23]. Moreover, it asserts that when people perceive themselves and others in terms of shared social identity, this provides the basis for a range of important group and organizational behaviors [24]–[28]. One of these is leadership. In line with this claim, a large body of research has shown that it is leaders' capacity to be perceived to advance the interests of a social identity that is shared with followers that enables them to secure support for their vision and motivate others to help turn it into reality [29]. Such analysis suggests that leaders are successful not because they demonstrate their individual superiority or because they think and act in terms of ‘I’, but rather because, and to the extent that, they are perceived to think and be acting in terms of the collective ‘we’.

Speaking to these claims, empirical evidence indicates that leaders’ increased social identification with a collective (i.e., the degree to which they have internalized the collective as part of their sense of self) is positively related to followers' favorable reactions to them [30]–[32]. Along similar lines, experimental studies have shown that when leaders use more we-referencing language followers are more likely to see them as charismatic [33]. Consistent with the idea that we-referencing language proves helpful to leaders outside the laboratory, there is also evidence that in the United States over the last two centuries references to the collective entities ‘we’, ‘people’ and ‘America’ have increased substantially in both State of the Union and Presidential inaugural addresses [34], [35].

However, prior research that has explored these ideas has tended to hone in selectively on exceptional addresses or on the oratory of particularly successful leaders (e.g., those in high political office) [2], [4], [7]. As a result, it is unclear whether we-referencing language is something that is broadly associated with, and predictive of, leaders' future success. More generally, it is unclear exactly how widespread such strategies are and there are questions about whether effects produced in laboratory studies of undergraduate students are applicable to the cut-and-thrust of leadership in the world at large.

### The Present Study

In order to address these lacunae, we sought to discover whether there is any more compelling evidence that political leaders' use of collective pronouns has a concrete bearing on their success. One resource that we identified as having the potential to prove useful for this purpose is recently released digitized transcripts of all the official campaign speeches made by leaders of the two major political parties for all general elections held in Australia since the creation of the Federal Parliament in 1901 [36]. This provided us with an opportunity to examine whether leaders' use of we-referencing (vs. I-referencing) language was a predictor of subsequent election victory. Whereas classical leadership models might lead one to expect that leaders who communicate a strong sense of their personal identity (through references to ‘I’ and ‘me’) would be more successful [13], the social identity approach leads us to predict that success would be more likely to follow from leaders' invocation of shared group identity in their speeches (through their use of ‘we’ and ‘us’).

## Methods

### Sample

Data were drawn from the official campaign speeches of successful and unsuccessful Prime Ministerial candidates representing the two major parties in all 43 Australian Federal elections since independence from Britain in 1901. These speeches are typically delivered about a month before the election. They are key events, widely reported in the media, in which leaders present their manifesto to the public at large. These speeches are also historical records that speak to national concerns at a particular point in time.

Speeches were included from every candidate up to and including the Federal Election in 2010 (*N* = 84; with the exceptions that George Reid did not deliver a speech in 1901 and that for Andrew Fisher's speech in 1910, the only available transcript is a press report that is written in third-person singular and does not contain direct speech). The first of these was the address delivered by Edmund Barton in 1901; the most recent were those delivered by Julia Gillard and her unsuccessful opponent Tony Abbott in 2010.

### Procedure and Analysis

For each speech we coded whether or not candidates were successful (i.e., whether they became Prime Minister or opposition leader) as well as the number of first-person singular (‘I’, ‘me’) and first-person plural pronouns (‘we’, ‘us’) that were used. All references to ‘I’ and ‘me’ as well as to ‘we’ and ‘us’ within a speech were combined to obtain indicators for the use of personal and collective pronouns, respectively. The total number of words in each speech was also included as a control variable.

As well as this, two independent raters read through every speech with a view to identifying the group that was referenced by each mention of ‘we’ and ‘us’. Preliminary analysis indicated that there were three main referents: the nation (Australia), the government, and the speaker's political party. Coders identified the primary group that each mention of ‘we’ and ‘us’ referred to, and in cases of ambiguity, also the secondary referent. In a random sample of nine speeches (10%) coders agreed on 85% of primary categorizations, and so the preponderance of primary referents was calculated by averaging across the two raters.

## Results

**Figure 1:**
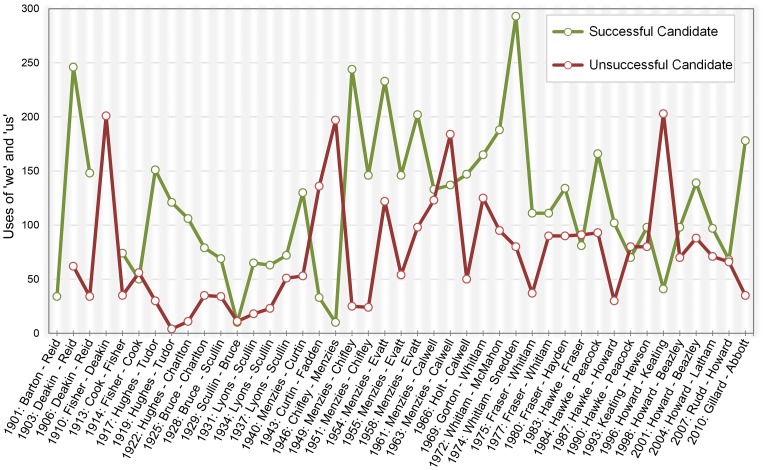
Use of collective pronouns (‘we’, ‘us’) by Australian Prime Ministerial candidates in election campaign speeches as a function of election year and candidates' success. Data for candidates representing the two leading parties; Election winner named first.

We conducted a binary logistic regression in order to examine whether candidates' election success was predicted by the number of times that they used personal (‘I’, ‘me’) and collective pronouns (‘we’, ‘us’) while also controlling for the absolute number of words in a given speech (see Table 1) [χ^2^ (3, *N* = 84) = 12.24, *p* = .007]. This analysis revealed a non-significant effect for speech length [*B* = –.01, *SE = *.26, *p = *.98, exp(*b*) = .99, 95% *CIs = *.59, 1.66] and use of personal pronouns ‘I’ and ‘me’ [*B = *.16, *SE = *.26, *p = *.54, exp(*b*) = 1.17, 95% *CIs = *.71, 1.93] (successful candidate: *M = *39.38, [*M*_I_ = 34.52, *M*_me_ = 4.86]; unsuccessful candidate: *M = *28.90, [*M*_I_ = 25.71, *M*_me_ = 3.19]). However, there was a significant effect for use of collective pronouns ‘we’ and ‘us’ [*B = *.81, *SE = *.30, *p = *.008, exp(*b*) = 2.24, 95% *CIs = *1.24, 4.06]. This exponential function of 2.24 points to a 124% increase in the odds of winning an election for an additional 63 references to ‘we’ and ‘us’ (equal to one standard deviation of the sample mean). As shown in Figure 1, in 80% of all elections (in 33 of 41 analyzable elections), successful candidates used more collective pronouns (*M = *118.79, [M_we_ = 106.93, *M*_us_ = 11.86]) than unsuccessful candidates (*M = *73.64, [*M*_we_ = 66.40, *M*_us_ = 7.24]).

**Table 1:** Logistic regression statistics as well as means and standard deviations displaying number of personal and collective pronouns used in election campaign speeches by unsuccessful and successful Australian Prime Ministerial candidates since 1901.

Variable	*Means* (*SD*) for unsuccessful candidates (*n* = 42)	*Means* (*SD*) for successful candidates (*n* = 42)	*B (SE)*	exp (*b*)	95% *CIs*
**Use of personal pronouns (‘I’, ‘me’)**	28.90 (25.24)	39.38 (33.11)	.16 (.26)	1.17	.71, 1.93
**Use of collective pronouns (‘we’, ‘us’)**	73.69 (52.42)	118.79 (64.75)	.81^**^ (.30)	2.24	1.24, 4.06
**Total no. of words in speech**	6813.93 (4184.17)	8.049.95 (3763.53)	–.01 (.26)	.99	.59, 1.66
			?^2^(3, *N* = 84) = 12.24^**^		
**No. of words per personal pronoun**	644.52 (1059.10)	556.90 (1212.77)	–.06 (.23)	.94	.60, 1.47
**No. of words per collective pronoun**	136.12 (139.54)	78.95 (48.39)	–.96^*^ (.42)	.37	.17,.87
			?^2^(2, *N* = 80) = 7.94^*^		

*Notes*: ^*^ *p*<.05. ^**^ *p*<.01. Variables for the logistic regression were *z*-standardized; Because two successful and two unsuccessful candidates used neither ‘I’ or ‘me’ in their speech, the sample sizes concerning the number of words per personal pronoun and thus the logistic regression results in the lower half of the table are reduced (*n = *40; *n = *40).

We also calculated the number of words in a speech per pronoun by dividing the total number of words by the number of times that personal pronouns (‘I’, ‘me’) and collective pronouns (‘we’, ‘us’) were used. Because two successful and two unsuccessful candidates used neither ‘I’ nor ‘me’ in their speech, sample sizes were reduced for this analysis [χ^2^ (2, *N = *80) = 7.94, *p = *.019]. Consistent with the patterns observed above, election success was not predicted by the number of words per reference to personal pronouns (‘I’, ‘me’) [*B = *–.06, *SE = *.23, *p = *.78, exp(*b*) = .94, 95% *CIs = *.60, 1.47], but it was by the number of words per reference to collective pronouns (‘we’, ‘us’) [*B = *–.96, *SE = *.42, *p = *.022, exp(*b*) = .38, 95% *CIs = *.17,.87]. This exponential function of.38 points to a 62% decrease in the odds of winning an election for an additional 108 words per use of ‘we’ or ‘us’ (equal to one standard deviation of the sample mean). Whereas unsuccessful candidates referred to ‘we’ or ‘us’ every 136 words (*SD = *139.54), successful candidates mentioned ‘we’ or ‘us’ every 79 words (*SD = *48.39). Testament to the robustness of these patterns, results were unaffected by the removal of outliers (with scores 3SDs above or below the sample mean).

Because one might argue that candidates’ election success is dependent on the rhetoric of the respective competitor, we also ran an additional analysis in which each election was treated as an independent event (rather than treating candidates as the unit of analysis). For this purpose, we examined the number of times that the successful candidate used personal (‘I’, ‘me’) and collective (‘we’, ‘us’) pronouns in their speech as a proportion of all personal and collective pronouns that were used by both them and the unsuccessful candidate in the same election (thus reducing the sample size to *N = *41). A one-sample *t*-test indicated that there was no difference in candidates’ use of personal pronouns [*t*(40) = 1.72, *p = *.09, *M*_Difference_ *= *7.42, 95% *CIs = *–1.28, 16.13], but a significant difference in their use of collective pronouns [*t*(40) = 4.36, *p*<.001, *M*_Difference_ *= *13.53, 95% *CIs = *7.25, 19.82] with successful candidates using the collective pronouns ‘we’ and ‘us’ significantly more often than unsuccessful ones.

Furthermore, we also tested whether the impact of we-referencing language depended on the ideology of the leader's party (e.g., being more effective for Labor than Liberal leaders). If party ideology (relatively progressive vs. relatively conservative; coded as –1 and 1, respectively) as well as the interaction between use of collective pronouns and party ideology are added as predictors of election success, the only effects were for party ideology (as conservative parties had been successful in 29 of all elections) [*B = *.52, *SE = *.25, *p = *.036, exp(*b*) = 1.68, 95% *CIs = *1.04, 2.73] and the use of we-referencing language [*B = *.73, *SE = *.31, *p = *.019, exp(*b*) = 2.08, 95% *CIs = *1.13, 3.84]. The interaction between ideology and use of we-referencing language was not significant [*B = *.22, *SE = *.29, *p = *.45, exp(*b*) = 1.24, 95% *CIs = *.71, 2.18]. Similar analysis examining the impact of party ideology together with words per personal pronoun, and words per collective pronoun on electoral success revealed an identical pattern. Thus the interaction between party ideology and words per ‘we’ and ‘us’ was not significant [*B = *.57, *SE = *.58, *p = *.34, exp(*b*) = 1.76, 95% *CIs = *.57, 5.44] and the only significant predictors were party ideology [*B = *.75, *SE = *.29, *p = *.011, exp(*b*) = 2.11, 95% *CIs = *1.18, 3.75] and words per ‘we’ and ‘us’ [*B = *–.92, *SE = *.46, *p = *.043, exp(*b*) = .40, 95% *CIs = *.16,.97]. In sum, the relationship between we-referencing language and election success appears to be independent of party ideology.

Analysis of the primary group that was referred to in each mention of ‘we’ and ‘us’, indicated that in 46.4% of cases this was the speaker’s political party, in 26.4% of cases it was the nation (Australia), in 25.5% of cases it was the government, and in just 1.7% of cases it was some other group (e.g., parents, people in general). Examples of references to each of these different groups are presented in Table 2 together with the total number of references made by successful and unsuccessful candidates.

**Table 2:** Who is this ‘we’?

Primary referent	Examples	*N* Uses by successful candidates	*N* Uses by unsuccessful candidates	*N* Total uses
**Nation**	“We need workers with hand, heart and head if we are to become a great nation.” (Robert Menzies, 1954)	1337	798	2135
	“We have more sheep than any other country in the world; and those, thanks to the enterprise, foresight, and patriotism of those engaged in the industry, produce the best merino wool in the world.” (Billy Hughes, 1922)	(62.6%)	(37.4%)	
**Government**	“We must address the ageing of our population and we must balance our need for economic growth with protection of our precious environment.” (John Howard, 2004)	1670	389	2059
	“Fellow Australians, we have fulfilled the fundamental pledge we made to you twenty-one months ago — to bring Australians together in the great task of recovery from the economic crisis which then confronted our nation.” (Bob Hawke, 1984)	(81.1%)	(18.9%)	
**Political party**	“Unlike Labor, we know that governments have no money of their own to spend — only taxpayers' money.” (Malcolm Fraser, 1980)	1891	1857	3748
	“The Labor Party's policy is constructive. We are not out to destroy, but to build up.” (James Scullin, 1934)	(50.5%)	(49.5%)	
**Other**	“For me, that's not just a policy. It's a personal commitment. Janine and I have got a great, big mortgage. We live in a mortgage belt street. We come from a mortgage belt community.” (Mark Latham, 2004)	91	51	142
	“As individuals, we only get what we can pay for.” (Joseph Lyons, 1937)	(64.1%)	(35.9%)	
**Total**		4989	3095	8084
		(61.7%)	(38.3%)	

Primary referent, examples, and uses of “we” and “us” in official election campaign speeches by successful and unsuccessful Australian Prime Ministerial candidates since 1901.

*Notes*: Because George Reid did not deliver an election campaign speech in 1901 and because to date no transcript has been located for Andrew Fisher's election campaign speech in 1910, the overall sample size is reduced to *N = *84. Percentages of total count within category are indicated in parenthesis.

## Discussion

The present findings extend upon previous work in at least three important ways. First, they suggest that leaders' use of we-referencing language is related to a very significant index of leader effectiveness — namely their capacity to marshal follower support that propels them to the highest office in the land [10], [35]. In this way, the findings complement, but also expand upon, prior research that has relied on laboratory-generated data to show that leaders' use of collective language enhances perceptions of their charisma [33]. Moreover, it also extends upon prior work showing that those political leaders who are seen to be charismatic make greater use of collective language [4]. By demonstrating that successful election candidates are more likely to use language that is rooted in the collective ‘we’ and ‘us’ than those who are unsuccessful, the research thus suggests that we-referencing language is far more than merely a hollow tool. For while cues that speak to a shared sense of ‘us’ may be subtle and often go unnoticed, they appear to be a powerful marker of leaders' future success.

Second, these findings support prior research which suggests that leaders act as *entrepreneurs of identity* who, through their rhetoric, actively seek to craft a sense among followers — both within their own party and beyond it — that they are part of the same group [6]–[9]. However, prior research has largely involved scrutinizing leaders' identity rhetoric by means of qualitative, discursive analysis and has tended not to incorporate quantitative analysis. The present paper fits nicely with these notions by providing, to our knowledge, the first quantitative demonstration from the field of the strong association between leaders' we-referencing language and their actual success. In this way, these findings suggest that entrepreneurs of identity not only define what ‘we’ and ‘us’ stand for (in terms of norms and values) but also strengthen their connection and ‘one-ness’ with potential followers through their heightened use of collective language. Indeed, in this regard, it is notable that in successful speeches candidates typically segue constantly between the narrow ‘us’ of their political party and/or the government and the broader ‘us’ of the nation — and much of their success can be attributed to their ability to make both forms of reference convincingly. This is seen, for example, in Gough Whitlam's 1974 election speech (which contained the highest number of references to ‘we’ and ‘us’; *N* = 293) where he commented:

“Through our economic policies and our social security program, Australia's prosperity is becoming more fairly shared than ever before. Abroad, Australia has never stood so tall. We have buried old animosities. We are held in new respect by old friends and allies.”

Third, by demonstrating that it is the ‘we’ and ‘us’ in a leader's talk (and not the ‘I’ and ‘me’) that distinguishes successful political leaders from their unsuccessful counterparts, the findings indicate that it is those leaders who think and act in terms of the collective (rather than in terms of themselves as individuals) who are most capable of mobilizing follower support for their leadership [18], [31]–[33]. In this, the findings support theoretical claims that followers do not support leaders because those leaders present themselves as standing above and *apart from* the group (i.e., as the great ‘I’) [11], [13] but rather because they present themselves as *a part of* the group (i.e., as a great ‘we’). At the same time, the fact that references to the collective are more frequently found in the rhetoric of victorious candidates seems likely to also reflect the fact that it is those leaders who are more confident that they are able to speak on behalf of groups defined at different levels of abstraction (party, government, nation) who are ultimately more likely to be given the opportunity to do so in future.

The present research is not, of course, without limitations. In particular, while an archival study has the advantage of relying on actual field data (rather than on data from contrived experiments with undergraduate students), it is limited in its capacity to provide insight into the psychological processes that are at play here. For instance, although the present research provides strong evidence for a prospective association between leaders' we-referencing language and their election victory, it cannot disentangle the relative importance of active, constructive versus reflective elements of this relationship. Nevertheless, we believe that this relationship is likely to be grounded in the two-way process discussed above. That is, on the one hand, we suggest that this association arises from the fact that leaders actively construe follower endorsement through their use of we-referencing language (in line with the temporal sequence and the prospective study design whereby election campaign speeches were given prior to federal elections). On the other hand, to the extent that leaders are aware of their popularity among followers at the time of giving their speeches, another significant part of this association is likely to be rooted in a reflective process. That is, it seems likely that leaders who feel themselves to be supported by, and representative of, followers (both within and beyond their party and government) are more likely to feel authorized to invoke a sense of the collective in their language.

Similarly, the relationship between leaders' use of we-referencing language and election success was found to be independent of party ideology. Nevertheless, it would be worth investigating whether there are meaningful variations in the strength of this relationship in other organizational and national contexts — for example, those differentiated in terms of geography, culture, and domain. Along these lines, future research might also investigate the ways in which this relationship varies as a function of (a) followers' perceived ideological differences with a leader or (b) the broader national context in which the speech is delivered (e.g., the degree of threat by other nations or the perceived severity of national crisis).

At the same time, though, we believe that there are two particular strengths to the present analysis that are worth underscoring. First, by offering a thoroughgoing examination of a very large number of leaders' speeches it goes beyond previous research that has tended to examine only selected (and limited) samples of leader rhetoric within any given domain. Second, by examining speeches that pertain to the highest political office in the land, it focuses on what is clearly a very important domain of leader activity. Indeed, although it may come in many different forms, leadership does not get much more significant than this.

## Conclusion

The present findings support claims that leaders' success arises from their capacity to speak (and be perceived to speak) on behalf of the groups whose members they are seeking to influence and to mobilize [20]–[22]. Indeed, they suggest that, for politicians at least, invoking — and being able to invoke — the collective ‘we’ and ‘us’ in one's overtures to potential followers is predictive of the all-important difference between victory and defeat. In this, the findings give empirical substance to John Adair's [15] observation that the most useful word in the leader's vocabulary is ‘we’, and the least useful word is ‘I’.
